# Description of *Harpagorhynchus golvaneuzeti* n. gen. n. sp. (Acanthocephala, Harpagorhynchinae n. sub-fam.) with a review of acanthocephalan parasites of soleid fishes in the Mediterranean Basin

**DOI:** 10.1051/parasite/2019012

**Published:** 2019-03-08

**Authors:** Yuriy Kvach, Isaure de Buron

**Affiliations:** 1 Institute of Marine Biology, National Academy of Sciences of Ukraine Pushkinska 37 65011 Odessa Ukraine; 2 Department of Biology, College of Charleston Charleston SC 29412 USA

**Keywords:** Acanthocephala, Palaeacanthocephala, Echinorhynchidae, Soleidae, *Solearhynchus*, Mediterranean basin

## Abstract

A species of acanthocephalan new to science from soleid fishes in the Mediterranean Sea and the Sea of Marmara is described. The new species is characterised by individuals having a club-shaped proboscis armed with 12–13 rows of 6–7 rooted hooks of a single type, a basal cerebral ganglion, and tegumental spines on the anterior two thirds of the body. Males have six cement glands and females show spines around the genital opening. To accommodate this species, a new genus, *Harpagorhynchus* n. gen., and a new subfamily in Echinorhynchidae, Harpagorhynchinae n. sub-fam., are erected. A critical review of the literature on echinorhynchid species infecting soleid fishes in the Mediterranean basin showed that *Solearhynchus soleae* (Porta, 1906) should be considered a junior synonym of *S. rhytidotes* (Monticelli, 1905) and that *S. kostylewi* (Meyer, 1932) is a valid species. An identification key of acanthocephalans of Mediterranean soleids is provided.

## Abstract (fr)

Une nouvelle espèce d’acanthocéphale de poissons soléidés de la Mer Méditerranée et de la Mer Marmara est décrite. Cette nouvelle espèce est caractérisée par des individus ayant un proboscis ovoïde portant 12 à 13 rangées de 6 à 7 crochets d’un seul type et avec racine vraie, un ganglion cérébroïde au fond du réceptacle, et un tronc couvert d’épines cuticulaires sur ses deux tiers antérieurs. Les mâles ont six paires de glandes cémentaires et les femelles des épines génitales. Un nouveau genre, *Harpagorhynchus* n. gen., et une nouvelle sous famille, Harpagorhynchinae n. subfam., sont proposés pour attribuer une position taxonomique à cette nouvelle espèce. Une revue critique de la littérature sur les espèces d’échinorhynchidés qui infectent les poissons soléidés dans le Bassin Méditerranéen démontre que l’espèce *Solearhynchus soleae* (Porta, 1906) est un synonyme junior de *S. rhytidotes* (Monticelli, 1905) et que l’espèce *S. kostylewi* (Meyer, 1932) est valide. Une clé de détermination des acanthocéphales de poisons soléidés du Bassin Méditerranéen est incluse.

## Introduction

Soleid fishes (Actinopterygii: Soleidae), commonly referred to as soles, are important fishery species in the Mediterranean basin [8]. In this region, they are known to be infected by only three species of acanthocephalans: the arhythmacanthid *Acanthocephaloides propinquus* (Dujardin, 1845) [10] was reported from the Adriatic sole *Pegusa impar* (Bennett, 1831) and the blackhand sole *P. nasuta* (Pallas, 1814) in various regions, including along the French coast [13], the Sea of Marmara [18], and the Black Sea [22]; and two species of the echinorhynchid *Solearhynchus* de Buron and Maillard, 1985 (*S. soleae* (Porta, 1906) [21] and *S. kostylewi* (Meyer, 1932) [15]) are known in three sole species [common sole *S. solea*, blackhand sole *P. nasuta* and sand sole *Pegusa lascaris* (Risso, 1810)] from the Mediterranean Sea [7], the Black Sea [4], and the Sea of Marmara [14].

While this diversity is quite low, it is important to note that the family Echinorhynchidae Cobbold, 1879 [9], which includes two sub-families (Echinorhynchinae Cobbold, 1879 [9] and Circinatechinorhynchinae Bhattacharya, 2007 [5]) and eight genera [1, 12], consists of numerous acanthocephalan species whose taxonomic status is at times unclear. In particular, *Echinorhynchus* Zoega in Müller, 1776 [17] is a genus that has been erroneously assigned to numerous acanthocephalans that, over the years, had then to be re-assigned to various genera (see list in [12]). In soleid fishes in the Mediterranean basin, there is still some confusion about *E. soleae* Porta, 1906 [21] and *E. rhytidotes* Monticelli, 1905 [16]. Both species were originally described from the Adriatic sole *P. impar* (see [16, 21]) and then reassigned to various genera [15, 20], only to be placed, albeit later considered erroneously, in *Solearhynchus* by de Buron and Maillard [7]. This latter re-assignment in turn generated confusion within the *Solearhynchus* [3, 4, 14], which we aim to clarify herein.

One of the authors (IB), along with J.-Y. Golvan (at the time Professor of Parasitology and Associate at the Museum National d’Histoire Naturelle, Paris, France), studied acanthocephalans from flatfishes from the French Mediterranean coast in 1982–1983, during which time they encountered an echinorhynchid different from previously described species. Description of this species was not published at the time, and a few decades later, YK was prompted by similar findings in the common sole from the Sea of Marmara to combine information from the two collections to provide herein the description of an echinorhynchid genus and species new to science. To accommodate this species, however, a new subfamily of Echinorhynchidae must be erected.

## Materials and methods

Common sole (*S. solea*) were sampled in the near-shore region of the Gulf of Lion near Sète (43°33′6310″N, 3°76′529″E), Mediterranean Sea, France, between July 1982 and August 1983. Fish were dissected within 24 h post capture. Acanthocephalans were collected from the intestine, placed in distilled water to induce evagination of the proboscis, and fixed in Alcohol-Formalin-Acetic acid (AFA). Specimens were then either mounted in Berlese or dehydrated in a series of alcohol, cleared in xylene, and mounted in Canada balsam. A total of 20 gravid females and 10 gravid and 5 immature males were studied morphologically. Measurements are provided as the mean followed by the range in parentheses. Holotype and paratypes were deposited at the Museum National d’Histoire Naturelle (MNHN), Paris, France. Additionally, three of 10 specimens (two males, one female) initially identified as *S. soleae* by Oğuz and Kvach [18] sampled from the common sole from Gemlik Bay, Sea of Marmara, were deposited (HWML 216145) in the collection of the Ataturk University in Erzurum, Turkey.

For the scanning electron microscopy (SEM) study, AFA fixed specimens were dehydrated in a series of ethanol, placed in acetone for 3 h, critical point dried, and coated with gold. Specimens were observed using a scanning electron microscope JSM 35.

The original descriptions and taxonomic revisions of parasites of soleids from the 19th century [16, 21] as well as the late 20th century [6, 7, 19], and the most recent data [3, 4, 14] were reviewed and critically analyzed.

## Results

### Harpagorhynchinae n. sub-fam.

urn:lsid:zoobank.org:act:642B7D30-AE47-4E88-84E3-75E7EDBD1821

Parasites of marine fishes. Body elongated. Trunk covered by tegument spines in anterior body in males and females. Genital spines in females only. Proboscis relatively short, armed with hooks of same type with true root. Cerebral ganglion at base of proboscis receptacle. Lemnisci claviform.

Etymology: This subfamily is named after Latin *harpago* to emphasize the presence of tegumental spines on all individuals and genital spines on females. Presence of an armed body is the essential character that differentiates this new sub-family from the Echinorhynchinae and the Circinatechinorhynchinae.

Type genus: *Harpagorhynchus* n. gen.

### *Harpagorhynchus* n. gen.

urn:lsid:zoobank.org:act:12B22719-66DA-469B-9D2B-12E84AB8329D

Body large, cylindrical. Proboscis club-shaped, relatively small. Hooks of one type, all with roots. Hook size increases progressively from apex towards middle of proboscis, then decreases towards base of proboscis. Neck small, two lateral sensory papillae. Trunk partially spinose with tegumental spines in both sexes and also genital spines in females. Male genital apparatus occupies less than half of posterior part of trunk. Säefftigen’s pouch well developed. Vaginal sphincter single. Genital pore terminal in males and sub-terminal in females.

### *Harpagorhynchus golvaneuzeti* n. sp. Figures 1, 2

urn:lsid:zoobank.org:act:97FBE1B7-398B-47E4-83C7-61CD8E9F3008

**Figure 1:**
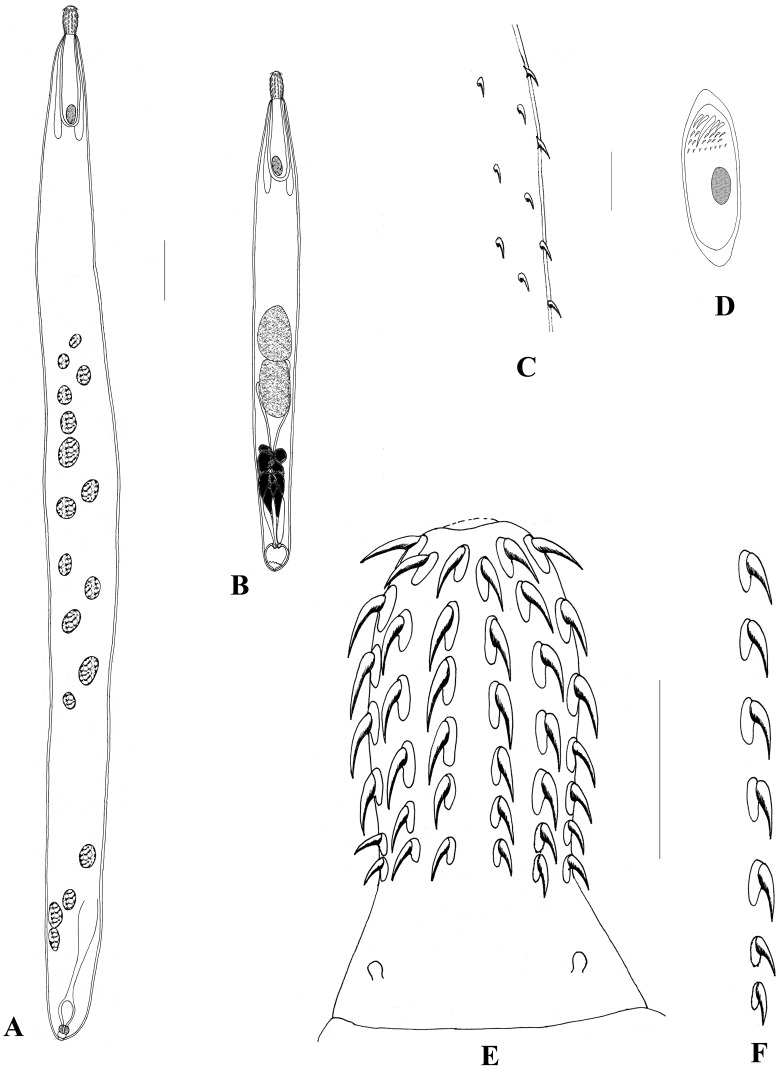
*Harpagorhynchus golvaneuzeti* gen. n. sp. n. A) Female total view; B) Male total view; C) Tegument with spines; D) Egg; E) Proboscis; F) A single hook row. Scales: A, B – 1 mm; C, D – 25 μm; E, F – 100 μm.

**Figure 2:**
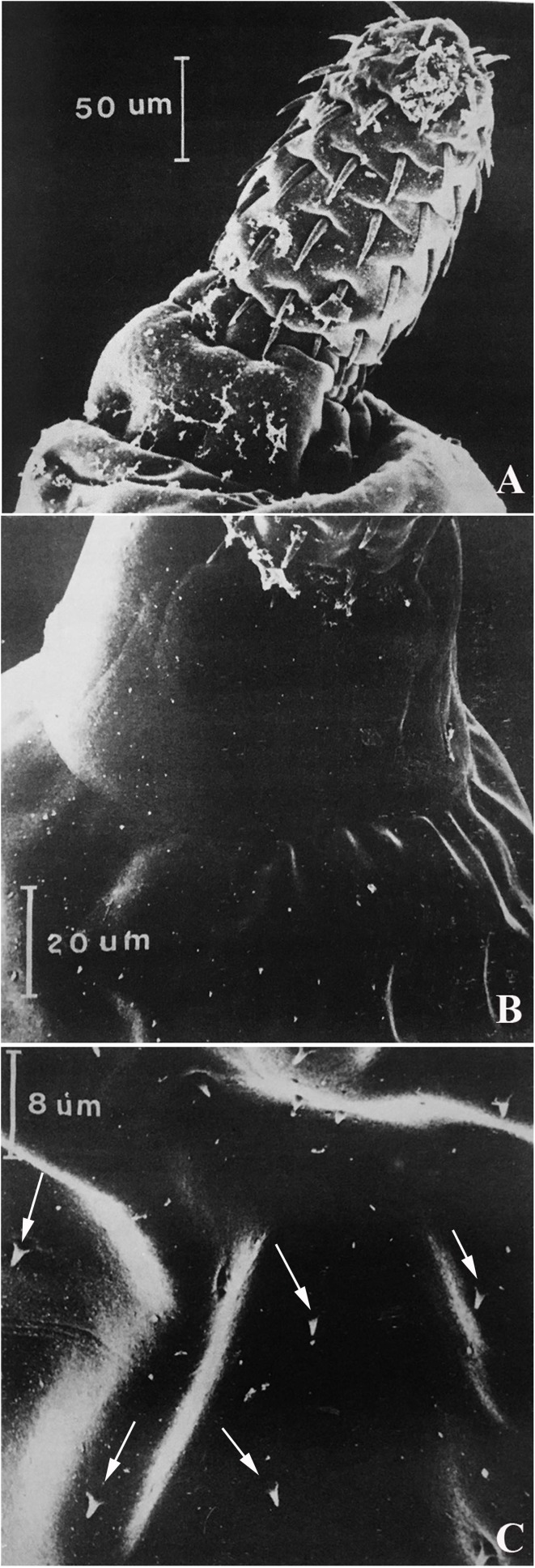
SEM photographs of *Harpagorhynchus golvaneuzeti* gen. n. sp. n. A) Proboscis; B) Neck with anterior part of trunk; C) Tegumental spines (arrows).

Type host: Common sole *Solea solea* (Quensel, 1806) (Soleidae, Pleuronectiformes)

Site of infection: Intestine

Other hosts: S*cophthalmus rhombus* (Linnaeus, 1758) (Scophthalmidae, Pleuronectiformes)

Type locality: Gulf of Lion near Sète, Mediterranean Sea, France

Other locality: Gemlik Bay, Sea of Marmara, Turkey

Collection date: 1982–1983 (France), 1990–1993 (Turkey)

Specimens deposited: Muséum National d’Histoire Naturelle, Paris, France, MNHN HEL755 (male holotype), MNHN HEL756 (female holotype), MNHN HEL753–MNHN HEL754 (paratypes).

Etymology: The specific epithet is proposed after Professors Yves-Jacques Golvan and Louis Euzet in their honor and in recognition of their mentorship to IB and their contribution to the description of this species when originally discovered. They are the ones who originally proposed *harpago* as a root for the subfamily and genus of this acanthocephalan species.

#### Description

Echinorhynchidae. Harpagorhynchinae. Sexual dimorphism noted only in size of worms. Body long, cylindrical, yellow to orange in color. Anterior part of body (about 2/3) covered with tegumental spines about 2 μm long, staggered, field pointed ventrally. Proboscis club-shaped, armed with 12–13 rows of 6–7 hooks each. Hooks all of same type, roots present. No abrupt difference in size of hooks, apical hooks ~35 μm long, middle hooks largest, ~45 μm long, posterior hooks smallest, 25 μm long. Root size decreases from apex to base of proboscis. Sensory papilla at tip of proboscis absent. Neck unarmed, short, 80 (60–100) μm long. Two sensory papillae on lateral sides of neck. Cerebral ganglion large, 200 (180–210) μm long × 90 (70–120) μm wide at base of double-walled proboscis receptacle. Two lemnisci of different lengths, both longer than receptacle.

Female body 12.5 (8–16) mm long, 0.85 (0.5–1) mm wide. Proboscis 265 (250–280) μm long, 130 (115–150) μm wide. Proboscis receptacle 680 (600–840) μm long, 170 (150–200) μm wide. Two lemnisci 1 (0.9–1.1) and 1.1 (0.9–1.25) mm long. Vaginal sphincter single, vulva ventral. Embryophore 80 μm long. Genital spines present, slightly larger than anterior tegument spines (about 2.5 μm long). Male body 6.5 (4.4–8.2) mm long, 0.8 (0.5–1) mm wide. Proboscis 245 (200–280) μm long, 130 (110–140) μm wide. Proboscis receptacle 620 (500–700) μm long, 150 (120–210) μm wide. Two lemnisci 1.05 (1–1.1) and 1.15 (1–1.3) mm long. Reproductive system occupies 40 (30–50)% of trunk. Testes in tandem, contiguous. Anterior 655 × 420 [(550 − 770) × (320 − 500)], posterior 570 × 400 [(450 − 750) × (300 − 500)]. Cement glands six, irregular, piriform with apical dilatations in 3–4 anterior glands. Säefftigen’s pouch 170 μm long. Six cement canals located on both sides of Säefftigen’s pouch. Genital pore terminal. Genital spines absent.

## Discussion

Based mainly upon the presence of six cement glands, a small neck, and one single type of hook on the proboscis, this species belongs to the family Echinorhynchidae, which currently comprises two valid sub-families: the Echinorhynchinae Cobbold, 1876 [9] and the Circinatechinorhynchinae Bhattacharya, 2007 [5]. The Echinorhynchinae has by far the largest number of species across a diverse set of fishes and whose individuals are all aspinose; the Circinatechinorhynchinae, on the other hand, is monospecific and contains individuals of *Circinatechinorhynchus pseudorhombi* Bhattacharya, 2007 [5] collected only from Indian marine fishes. This latter species is characterized by having circinate lemnisci encircling the junction of the proboscis and the proboscis receptacle and a last row of hooks much smaller than all others. Individuals of the newly described species are not Echinorhynchinae based on their spinose body and are not Circinatechinorhynchinae, as they clearly differ by having normal claviform lemnisci and hooks that do not change size abruptly. While the constitution of the Echinorhynchidae by these two subfamilies is the current classification, Golvan [13] had included worms “with faintly spinose trunk” in the Echinorhynchidae and erected the sub-family Yamagutisentinae to include *Yamagutisentis* Golvan, 1969 [13], the only genus in the Echinorhynchidae whose individuals had a spinose tegument. However, Yamagutisentinae was eliminated when Araki and Machida [2] synonymised *Yamagutisentis* with *Acanthocephaloides* (Family Arhythmacanthidae Meyer, 1932 [15]) because individuals had a proboscis armed with hooks of different types. Based on this reasoning, it is necessary to erect a new subfamily to place the species found in soleids, which will differ from the two current subfamilies in having members with a spinose tegument. We thus propose the following key for identification of the Echinorhynchidae sub-families:

1(2) Anterior part of trunk covered with spines in males and females, genital spines in females………….…………………………………........................................................ Harpagorhynchinae
2(1) Trunk aspinose......................................................................................................... 3
3(4) Lemnisci claviform, not very long........................................................................... Echinorhynchinae
4(3) Lemnisci circinate or a ring-like compact mass, encircling the junction of proboscis and proboscis receptacle......................................................................................................................... Circinatechinorhynchinae

*Harpagorhynchus golvaneuzeti* n. gen. n. sp. is the third echinorhynchid species known in soleid fishes from the Mediterranean Basin. The two other species, both from Europe, are representatives of the sub-family Echinorhynchinae: *S. soleae* and *S. kostylewi* [7, 14]. *Solearhynchus* was erected by de Buron and Maillard [7] to accommodate marine species with basal cerebral ganglia and these authors also synonymized *E. rhytidotes* at that time. However, because Porta’s description [21] of *S*. (*E.*) *soleae* was in fact published in 1906, and not in 1905 as regularly mentioned in the literature, *S*. (*E.*) *rhytidotes* has priority over *S*. (*E.*) *soleae*. It is, therefore, *S. rhytidotes* that must be considered a valid species, with *S. soleae* as a junior synonym. In support of this chronology, it is noteworthy that in the same 1906 publication, Porta [21] emended the description of *S*. (*E.*) *rhytidotes*, confirming without a doubt that *S. rhytidotes* was described before *S. soleae.* Furthermore, while Belofastova [4] argued that *Acanthocephaloides kostylewi* (later re-described as *S. kostylewi* [14]) is a junior synonym with *S. rhytidotes,* Gaevskaya [11] later did not support this idea, as *S. kostylewi* differs from *S. rhytidotes* by the number of longitudinal rows of hooks [14]. We therefore consider *S. kostylewi* as a valid species of the genus *Solearhynchus*.

We propose the following identification key of acanthocephalans from the soleid fishes in the Mediterranean Basin:

1(2) Proboscis armed by hooks of one type…………………………………………… 3
2(1) Proboscis armed by hooks of different types. Trunk covered with spines………. *Acanthocephaloides propinquus* Dujardin, 1845 (Arhythmacanthidae)
3(4) Trunk spinose – females with genital spines (Echinorhynchidae: Harpagorhynchinae n. sub-fam)................................................................................................................................. *Harpagorhynchus* n. gen. n. sp.
4(3)Trunk aspinose (Echinorhynchidae: Echinorhynchinae)......................................... 5
5(6)Proboscis armed with 12 rows of hooks of 5-6 each; eggs with four lateral-longitudinal swellings......................................................................................................................... *Solearhynchus rhytidotes* (Monticelli, 1905)
6(5) Proboscis armed with 16 rows of hooks of 5–6 each; eggs with polar prolongation……… ..................................................................................................................................... *Solearhynchus kostylewi* (Meyer, 1932)
